# Assessment of Correlation between Dual-Energy Ct (De-Ct)-Derived Iodine Concentration and Local Flourodeoxyglucose (Fdg) Uptake in Patients with Primary Non-Small-Cell Lung Cancer

**DOI:** 10.3390/tomography8040149

**Published:** 2022-07-08

**Authors:** Michael Brun Andersen, Aska Drljevic-Nielsen, Jesper Thygesen, Matthijs Ferdinand Kruis, Karin Hjorthaug, Finn Rasmussen, Jasper Albertus Nijkamp

**Affiliations:** 1 Department of Radiology, Aarhus University Hospital, 8200 Aarhus, Denmark; askadrlj@rm.dk (A.D.-N.); finnrasm@rm.dk (F.R.); 2 Department of Radiology, Copenhagen University Hospital, 2100 Copenhagen, Denmark; 3 Department of Clinical Engineering, Aarhus University Hospital, 8200 Aarhus, Denmark; jesthy@rm.dk; 4 Clinical Science CT, Philips Healthcare, Veenpluis 4-6, 5684 PC Best, The Netherlands; matthijs.kruis@philips.com; 5 Department of Nuclear Medicine and PET, Aarhus University Hospital, 8200 Aarhus, Denmark; karin.hjorthaug@rm.dk; 6 Department of Clinical Medicine, Aarhus University, 8200 Aarhus, Denmark; 7 Danish Center for Particle Therapy, Aarhus University Hospital, 8200 Aarhus, Denmark

**Keywords:** computed tomography, ^18^F-Flourodeoxyglucose PET/CT, lung cancer

## Abstract

(1) The current literature contains several studies investigating the correlation between dual-energy-derived iodine concentration (IC) and positron emission tomography (PET)-derived Flourodeoxyglucose (^18^F-FDG) uptake in patients with non-small-cell lung cancer (NSCLC). In previously published studies, either the entire tumor volume or a region of interest containing the maximum IC or ^18^F-FDG was assessed. However, the results have been inconsistent. The objective of this study was to correlate IC with FDG both within the entire volume and regional sub-volumes of primary tumors in patients with NSCLC. (2) In this retrospective study, a total of 22 patients with NSCLC who underwent both dual-energy CT (DE-CT) and ^18^F-FDG PET/CT were included. A region of interest (ROI) encircling the entire primary tumor was delineated, and a rigid registration of the DE-CT, iodine maps and FDG images was performed for the ROI. The correlation between tumor measurements and area-specific measurements of IC_peak_ and the peak standardized uptake value (SUV_peak_) was found. Finally, a correlation between tumor volume and the distance between SUV_peak_ and IC_peak_ centroids was found. (3) For the entire tumor, moderate-to-strong correlations were found between SUV_max_ and IC_max_ (R = 0.62, *p* = 0.002), and metabolic tumor volume vs. total iodine content (R = 0.91, *p* < 0.001), respectively. For local tumor sub-volumes, a negative correlation was found between IC_peak_ and SUV_peak_ (R = −0.58, *p* = 0.0046). Furthermore, a strong correlation was found between the tumor volume and the distance in millimeters between SUV_peak_ and IC_peak_ centroids (R = 0.81, *p* < 0.0001). (4) In patients with NSCLC, high FDG uptakes and high DE-CT-derived iodine concentrations correlated on a whole-tumor level, but the peak areas were positioned at different locations within the tumor. ^18^F-FDG PET/CT and DE-CT provide complementary information and might represent different underlying patho-physiologies.

## 1. Introduction

In spite of recent advances in treatment, lung cancer continues to be the leading cause of cancer-related deaths worldwide [1]. Imaging plays a pivotal role in the diagnosis, staging, therapy planning, response assessment and management of these patients. The primary imaging modality is contrast-enhanced computed tomography (CE-CT), as it provides excellent morphological assessment of the primary tumor, involvement of mediastinal lymph nodes and most sites of distant metastases. However, conventional CE-CT does not provide any functional information about tumor metabolism, perfusion or physiology [2,3]. Therefore, in NSCLC, it is often combined with 18-Fluorodeoxyglucose positron emission tomography (^18^F-FDG PET/CT) in order to provide information about glucose metabolism for staging [4]. Other imaging techniques, such as dynamic-contrast-enhanced CT (DCE-CT) and dual energy CT (DE-CT), can be used to quantify perfusion parameters and iodine concentration, respectively [5,6,7,8,9]. Although these imaging modalities represent different aspects of tumor physiology, previous studies have suggested a positive correlation between perfusion parameters, FDG uptake and iodine concentration [10,11].

There are some inconsistencies in the current literature on this topic. Miles et al. showed an inconsistent relationship between the standardized perfusion value and standardized uptake value (SUV) dependent on tumor size. This study was comparable to the results of Tateishi et al., which showed a correlation between peak attenuation, relative flow and FDG uptake. Contrarily, van Elmpt et al. found no direct correlation between DCE-CT parameters and FDG uptake [10,12,13,14].

Based on the correlation between iodine uptake and perfusion parameters, some studies have hypothesized that a correlation between DE-CT parameters and FDG uptake is plausible [15]. Similar to studies on DCE-CT parameters, there are discrepancies in the current literature. The studies of Schmid-Bindert et al. and Baxa et al. found a strong correlation between SUV_max_ and DE-CT parameters in patients with non-small-cell lung cancer (NSCLC), whereas the studies of Kupik et al. and Aoki et al. found no correlation regardless of tumor histology, but a moderate negative correlation for small primary tumors (<3 cm) [11,16,17,18].

In all previous studies, either a small region of interest (ROI) or the entire tumor volume was assessed. As NSCLC often exhibits high intra- and intertumoral heterogeneity, assessment of a small region of the tumor is a limitation [11,16,17,18].

The aim of the present study was to investigate the correlation between ^18^F-FDG PET/CT parameters and DE-CT-derived iodine concentrations for the total volume of primary lung tumors. In addition, the same correlation was assessed at the level of sub-volumes of the tumor.

## 2. Materials and Methods

### 2.1. Design and Patients

This study was retrospective, following the STROBE guidelines [19], including all patients with NSCLC who had undergone both DE-CT and ^18^F-FDG PET/CT between June 2016 and July 2017 at Aarhus University Hospital. In Denmark, it is standard for patients with NSCLC that can potentially undergo curative treatment to receive both a contrast-enhanced CT and an ^18^F-FDG PET/CT. Inclusion criteria: histologically verified NSCLC, written informed consent for the use of image and patient data and a period of less than 60 days between DE-CT and ^18^F-FDG PET/CT. Furthermore, to ensure a minimal difference of the tumors between the DE-CT and ^18^F-FDG PET/CT, both examinations should have been performed prior to the initiation of treatment.

### 2.2. Contrast-Enhanced DE-CT Imaging Protocol

CT imaging was performed with a multiple-row dual-layer detector CT scanner (Philips IQon CT; Philips Healthcare, Best, The Netherlands). CT acquisition parameters were 64 × 0.625 mm collimation, kV 120–140, mAs/slice 150–250, rotation time 0.75 s, reconstruction thickness 2 mm, increment 1 mm, pitch 1.078, FOV 35 cm and matrix 512 × 512. Computed tomography (CT) examinations included the chest and upper abdomen. Iodixanol 320 mg/mL (Visipaque^®^ 320; GE Healthcare, Boston, MA, USA) was injected intravenously at weight-adjusted doses of 1.7 mL/kg body weight to adjust for differences in distribution volume, and with an injection rate of 4 mL/s. A bolus tracking technique with an ROI in the descending aorta at the level of carina was used to adjust for differences in cardiac output. CT was performed during inspiration breath hold after a delay of 15 s for the chest and upper abdomen (late arterial phase), and 50 s for the upper abdomen (portovenous phase) after a threshold of 150 HU was obtained according to a standardized protocol at our institution. The arterial phase was chosen for assessment, as it covered the entire chest. From the scan, we obtained a contrast-enhanced CT scan reconstruction (CE-CT), and an iodine concentration (IC) map, providing the IC in mg per ml in each voxel.

### 2.3. _18_F-FDG PET/CT Imaging Protocol

Whole-body ^18^F-FDG PET/CT was performed with an integrated PET/CT scanner (Siemens Biograph w. 64-slice CT scanner; Siemens Healthcare, Forchheim, Germany). Participants were instructed to fast for 6 h prior to the examination. Approximately 400 MBq FDG was injected intravenously. ^18^F-FDG PET/CT scans were performed after a delay of 60 min. The FDG PET acquisition was performed with respiratory gating on inspiration, and the images were corrected for scatter and iteratively reconstructed. The nonenhanced CT acquisition parameters were: 120 kVp, 50 mAs, CTDIvol 3 mGy, 24 × 1.2 mm collimation. Reconstruction parameters: 3.0 mm section thickness, and 1.5 mm increment. The CT scan was acquired with inspiration breath hold.

### 2.4. Delineation of Lesions and Image Assessment

An expert in thoracic radiology with 12 years’ experience performed all delineations of the primary tumors. The radiologist was blinded to patient identifiers, clinical data and tissue sampling. Delineations were performed semi-automatically using Intellispace v-11, Tumor Tracking (Philips Healthcare, Best, The Netherlands). An ROI including the total circumference of the primary lung tumor was delineated on the CE-CT. Visible blood vessels within the tumor were excluded from the ROI. The ROIs were saved as RT structure sets and transferred for further analysis.

### 2.5. Data Processing

*Image registration:* In-house developed software was used to register the imaging data. First, the CT scan from the PET was rigidly registered to the CE-CT scan. Correlation ratio was used as metric, only taking the tumor region plus a 1 cm margin into account. Both translations and rotations were used. After visual inspection of the CT registration, the transformation matrix was also applied to the PET scan. Again, visual inspection was performed, and manual adaptation of the registration was allowed to correct for any residual mis-matches which could have been caused by differences in the inspiration breath hold in the different imaging modalities.

*Data analysis:* The imaging data were cropped to the tumor volume plus a 1 cm margin, and all scans were resampled to a 1 mm uniform grid, and exported in nifty format. PET scans were first converted from raw counts to standard uptake values (SUVs). Data analysis was performed in python 3.7 (Python Software Foundation). For each tumor, the following parameters from the PET were calculated: SUV_max_, SUV_mean_, metabolic tumor volume (MTV = SUV > 2.5), MTV_50%_ (volume at the threshold of 50% of SUVmax), MTV_42%_, MTV_30%_, MTV_20%_, MTV_10%_; total lesion glycolysis (TLG = MTV × SUV_mean_) for all the different MTV threshold levels (TLG_SUV2.5_, TLG_50%_, TLG_42%,_ TLG_30%,_ TLG_20%,_ TLG_10%_) and SUV_peak_ (highest average SUV for a 1 cm^3^ spherical ROI within the tumor).

Based on the IC, we calculated the following parameters from the tumor region: Iodine_max_, Iodine_mean_, total iodine content (TIC = tumor volume × Iodine_mean_), Iodine_peak_ (highest average iodine concentration for a 1 cm^3^ spherical ROI within the tumor similar to what was used for SUV_peak_), Iodine_max_@SUV_peak_ROI (maximum iodine concentration within the ROI used for the SUV_peak_).

Finally, we calculated the tumor volume based on the voxels included in the tumor ROI, and the distance between the centroid of the SUV_peak_ ROI, and the centroid of the Iodine_peak_ ROI.

### 2.6. Statistical Analysis

All PET parameters were correlated with the iodine parameters using Pearson’s correlation coefficient. QQ plots were used to test for normality. Parameters related to volume were not normally distributed, and therefore, a log^10^ transform of the data was used before calculating the correlation coefficient. Excel 2019 (Microsoft Corporation, Redmond, Washington, DC, USA) was used for the calculations, and *p*-values < 0.05 were deemed significant.

## 3. Results

### 3.1. Patients

**Figure 1:**
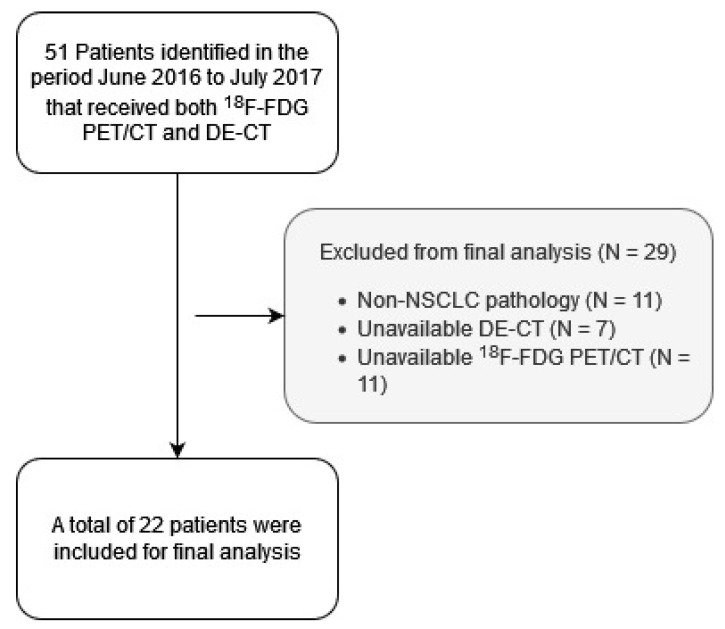
Flowchart showing patient inclusion/exclusion.

In total, 22 patients were included, 9 males and 13 females with a mean age of 73 years (range 64 to 92). The mean number of days between DE-CT and ^18^F-FDG PET/CT was 13 days (Table 1, Figure 1).

### 3.2. Correlation between Metabolic and Iodine-Related Parameters for the Whole Tumor

**Figure 2:**
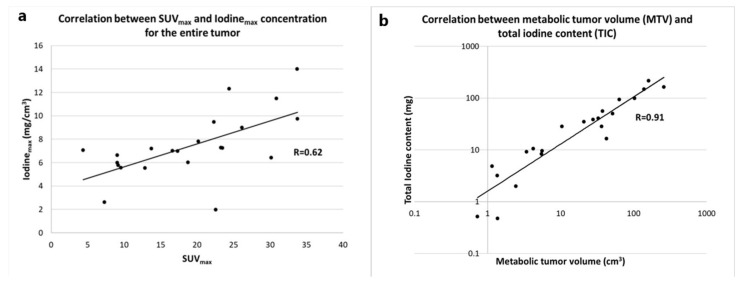
(**a**) Scatter plot showing the correlation between SUV_max_ in the tumor and maximum iodine concentration in the tumor. Data were fitted with a linear function, showing a significant correlation (*p* = 0.002). (**b**) Scatter plot showing the correlation between metabolic tumor volume (volume within the tumor with an SUV > 2.5) and total iodine content. Both axes are depicted on a log scale, because values were not normally distributed. Data were fitted with a power function, resulting in a significant correlation with *p*-value < 0.0001.

We found a positive correlation between SUV_max_ and Iodine_max_ (R = 0.64, *p* = 0.002) and a strong positive correlation between metabolic tumor volume (MTV) and total iodine content (R = 0.91, *p* < 0.0001), similar to previous studies (Figure 2) [11,17].

A total overview of the Pearson correlations between FDG uptake and iodine-related parameters is presented in Table 2.

### 3.3. Correlation with Tumor Volume

**Figure 3:**
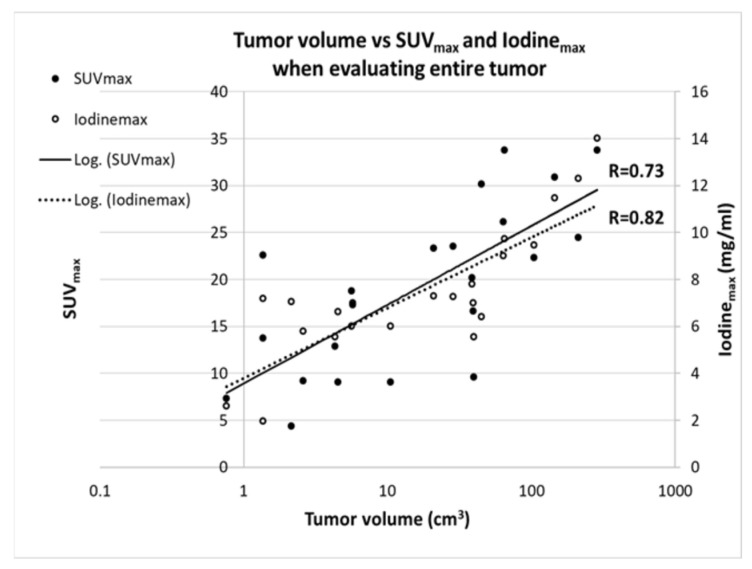
Scatter plot showing the correlation between tumor volume and SUV_max_ and the Iodine_max_. Tumor volume is depicted on a log scale, because values were not normally distributed. Data were fitted with a logarithmic function. Both logarithmic data fits showed a significant correlation (*p*-value SUV_max_ = 0.0001; Iodine_max_ = <0.0001).

For both SUV_max_ and Iodine_max_, we found a moderate positive correlation with tumor volume, which was relatively similar for both parameters (SUV_max_ R = 0.73, *p* = 0.0001; Iodine_max_ R = 0.82, *p* < 0.0001) (Figure 3).

### 3.4. Correlation of Max Iodine Concentration and SUV_peak_

**Figure 4:**
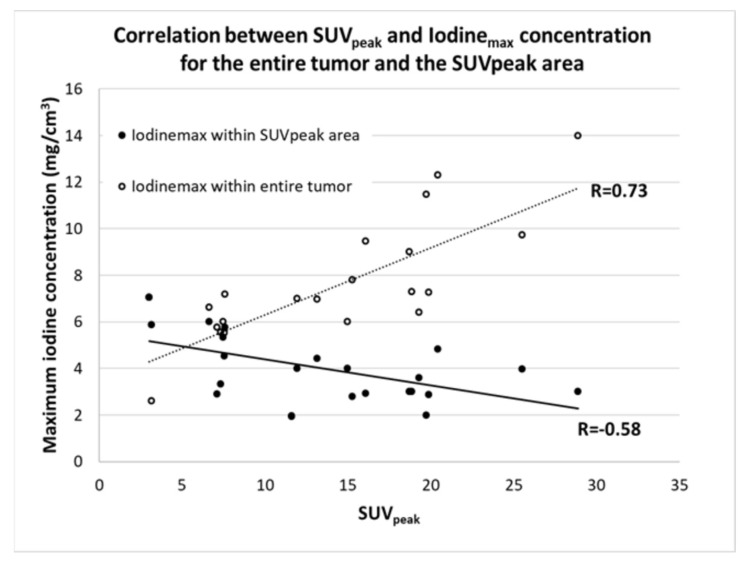
Scatter plot showing the correlation between the SUV_peak_ and the maximum iodine concentration (Iodine_max_) found in the tumor, and only in the SUV_peak_ area. There was a significant positive correlation (*p* = 0.0001) between the SUV_peak_ and the Iodine_max_ found in the tumor. However, when the maximum iodine concentration was taken from the same area as the SUV_peak_ (1 cm^3^ sphere), a significant negative correlation (*p* = 0.005) was found.

When correlating Iodine_max_ for the entire lesion with SUV_peak_, we found a moderate positive correlation (R = 0.73, *p* = 0.0001). However, when considering only the maximum concentration with the same area as the SUV_peak_ within a 1 cm^3^ spherical ROI, the correlation altered to a significant negative correlation (R =−0.58, *p* = 0.0046) (Figure 4).

**Figure 5:**
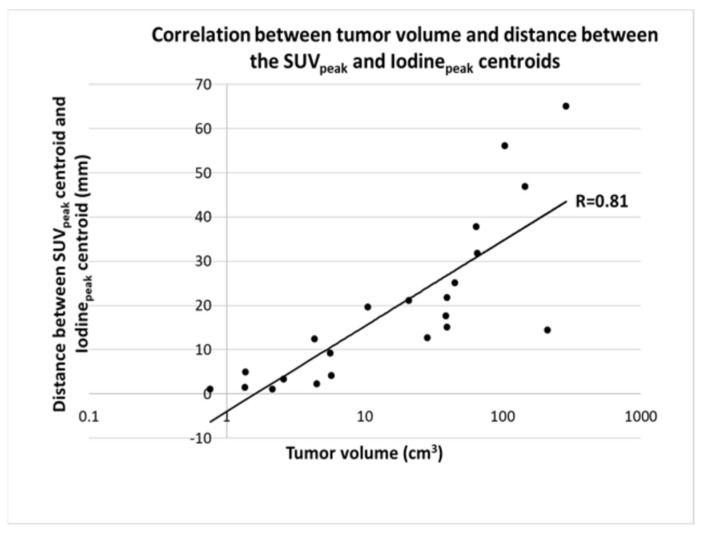
Scatter plot showing the correlation between the tumor volume (on a log scale) and the distance between the SUV_peak_ centroid and the Iodine_peak_ centroid. There was a significant positive correlation (*p* < 0.0001) between both, indicating that with an increase in tumor volume, there is an increase in the distance between the high-FGD-uptake area, and the high-iodine-uptake area.

**Figure 6:**
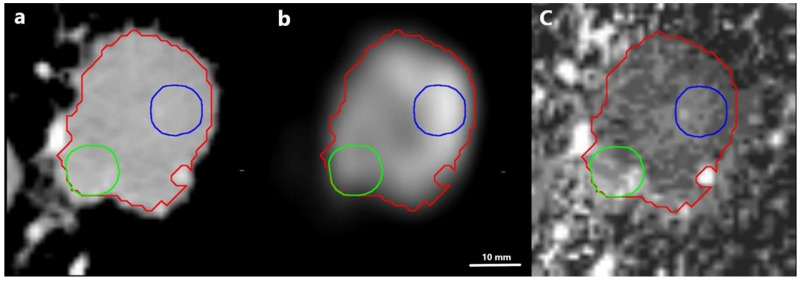
Illustration of the distance between the SUV_peak_ ROI (blue outline) and the Iodine_peak_ ROI (green outline) in a patient with a tumor of 21 cm^3^ (red outline). The distance between the two peak ROIs was 21 mm. (**a**): Iodine concentration map, (**b**): SUV map, (**c**): overlay of iodine concentration map and SUV map.

This is further illustrated by the correlation between the tumor volume and the distance between the SUV_peak_ centroid and the Iodine_peak_ centroid, showing a significant moderate positive correlation (R = 0.81, *p* < 0.0001) (Figure 5 and Figure 6).

## 4. Discussion

In this retrospective study, we found a moderate correlation between SUV_max_ and Iodine_max_ as well as a strong positive correlation between the MTV and total iodine content when considering the whole tumor. In addition, we also found a weak-to-moderate positive correlation between the tumor size and SUV_max_ and between the tumor size and Iodine_max_. Furthermore, when correlating the SUV_peak_ with the Iodine_max_ the correlation was dependent on what area the latter was measured in. When considering the entire tumor volume, a moderate-to-strong positive correlation was found, while when only a 1 cm^3^ sphere surrounding the SUV_peak_ was considered, the association changed to a moderate negative correlation. Finally, we found a moderate positive correlation of the distance between the centroid of SUV_peak_ and iodine_peak_ with the tumor volume.

Previous studies investigating the correlation between FDG uptake and iodine concentration all have a common denominator. The methodology in tumor delineation either included the entire tumor volume or a small ROI without registration between the PET scan and the IC images. In the study of Schmid-Bindert et al., the iodine parameters were measured by placement of three ROIs within the tumor; no specifics regarding the placement of these are described, and the mean and standard deviation of the measurements were subsequently recoded. The ^18^F-FDG PET-related measurements were based on an ROI encircling the entire tumor, including a 1 cm rim. Even though both data sets were available when the delineation was performed, it was performed by individual radiologists without a previous image registration between DE-CT and 18F-FDG PET/CT. In the study of Baxa et al., two radiologists performed the delineation of both the DE-CT and the ^18^F-FDG PET/CT using a semiautomatic approach encircling the entire lesion. However, similar to the study of Schmid-Bindert et al., no registration between the two modalities was performed. This may explain the discrepancies between the previous studies and our study. Baxa et al. and Schmid-Bindert et al. found a correlation between metabolic parameters and iodine-related parameters, which is in contrast to the results of Kupik et al. and Masahiko et al., who found no correlation. In addition, Kupik et al. found a moderate negative correlation for tumors <3 cm [11,16,17,18]. In this current study, we accounted for intratumoral heterogeneity by first performing an image registration between the DE-CT and the ^18^F-FDG PET/CT followed by a correlation of maximum iodine concentration with SUV_peak_ both within the entire tumor and in a sphere around the SUV_peak_. The results show that the maximum iodine concentration was located outside of the metabolic hotspot within the tumor, especially when the tumors became larger (Figure 4). This is further substantiated by the results showing an increase in the distance between SUV_peak_ and iodine peak locations in larger tumors (Figure 5). It is apparent that the metabolic activity and iodine quantification were located in different areas of the tumor, underlining that the physiological processes behind the parameters are not the same.

^18^F-FDG is a measurement for the metabolic activity. This has previously been correlated with high proliferation measured by the KI-67 index, suggesting the areas containing the SUV_peak_ are rapidly growing areas within the tumor [20]. As tumor cells are dependent on fast formation of new vessels by angiogenesis to supply the nutrients and oxygen needed to continue growth, the areas with a high proliferation will usually have poorly formed vessels with an inadequate basement membrane, causing them to leak [21]. Folkman et al. described how this increases the interstitial pressure within the tumor, and, combined with a relative absence of lymphatic vessels, it can cause vascular compression to some extent, prohibiting iodinated contrast from reaching the vessels [22]. In our study, we only had a late-arterial-phase DE-CT scan, which mainly represents blood flow and blood volume. The late-arterial-phase iodine concentration data likely show subsegments of tumor with a high degree of unobstructed normal vasculature that allow iodinated contrast to enter the area. This may be a potential explanation for the negative correlation between the SUV and iodine measurements within the SUV_peak_ area.

The primary limitation of this study was the small study population with only two squamous cell carcinoma patients; thus, a subgroup analysis on tumor type would not be meaningful. Secondly, there was a lack of histological and metabolomic correlation with our findings. A registration of the histological cross sections and metabolism of the tumor with high FDG and high iodine values could potentially explain the mechanisms behind our findings based on growth patterns, vessel density or vessel types and measurements of proliferation. Another limitation was the registration accuracy between the PET images and the SCE-CT quantitative images. As they were obtained at different points in time, and all had different protocols regarding breathing during the acquisition, we were not able to obtain a registration accuracy to the level where a voxel-by-voxel correlation could be performed. Furthermore, due to the retrospective design, there was a temporal difference between DE-CT and PET. Finally, this study was retrospective and a single-center study performed only on one platform for obtaining the iodine quantifications.

## 5. Conclusions

In conclusion, this feasibility study demonstrated a moderate correlation on the whole-tumor level between FDG uptake and iodine concentration. However, geographically high uptake of FDG and iodine was positioned in different areas of primary NSCLC tumors. Our findings suggest that underlying physiological traits responsible for iodine enhancement and FDG uptake in NSCLC are different and situated in separate tumor sub-compartments. 

## Figures and Tables

**Table 1:** Patient demographics including age, sex, days between PET and SCE-CT scans, tumor size, pathology, clinical stage and T stage.

Age	Years of Age	73 (CI95% 69.8 to 76.2)
Days between PET/CT and SCE-CT	Number of days	13 (CI95% 6.5 to 19.6)
Tumor size	Volume (cm^3^)	51.49 (CI95% 18.16 to 84.83)
Gender	Male	9
	Female	13
Pathology	Adenocarcinoma	19
	Squamous Cell Carcinoma	2
Clinical stage	IA	3
	IB	3
	IIA	1
	IIIA	3
	IIIB	1
	IIIC	4
	IVA	1
	IVB	6
T stage	T1a	0
	T1b	3
	T1c	1
	T2a	4
	T2b	3
	T3	5
	T4	6

**Table 2:** A summary of Pearson correlations of FDG uptake and iodine-related parameters at different thresholds of metabolic values with accompanying *p*-values. SUV: standard uptake value; MTV: metabolic tumor volume; TLG: total lesion glycolysis; r: Pearson correlation coefficient. All parameters that are related to volume (MTV; TLG; total iodine content) were log^10^-transformed to achieve normal distribution before calculating the correlation coefficient.

		Total Iodine Content		Iodine_mean_ (mg/cm^3^)		Iodine_max_ (mg/cm^3^)	
Parameter	Threshold	R	*p*-Value	R	*p*-Value	R	*p*-Value
SUV_max_		0.61	0.002	−0.53	0.012	0.62	0.001
SUV_peak_		0.68	0.001	−0.44	0.039	0.73	<0.001
SUV_mean_	50%	0.58	0.005	−0.51	0.015	0.60	0.003
	42%	0.57	0.006	−0.50	0.017	0.59	0.004
	30%	0.54	0.010	−0.47	0.027	0.57	0.006
	20%	0.48	0.025	−0.44	0.039	0.52	0.014
	10%	0.37	0.092	−0.39	0.070	0.42	0.051
MTV	50%	0.68	0.001	−0.30	0.168	0.70	<0.001
	42%	0.72	<0.001	−0.33	0.131	0.74	<0.001
	30%	0.80	<0.001	−0.37	0.088	0.80	<0.001
	20%	0.86	<0.001	−0.39	0.069	0.84	<0.001
	10%	0.92	<0.001	−0.40	0.064	0.86	<0.001
	SUV_2.5_	0.91	<0.001	−0.40	0.064	0.86	<0.001
TLG	50%	0.63	0.002	−0.31	0.158	0.69	<0.001
	42%	0.66	0.001	−0.33	0.137	0.72	<0.001
	30%	0.72	<0.001	−0.35	0.110	0.76	<0.001
	20%	0.75	<0.001	−0.36	0.097	0.78	<0.001
	10%	0.78	<0.001	−0.37	0.092	0.80	<0.001
	SUV_2.5_	0.77	<0.001	−0.37	0.012	0.80	<0.001

## Data Availability

The data supporting the reported results are not publicly available due to Danish data handling laws and the GDPR. However, upon request and procurement of a data handling agreement, the data can be shared/viewed in accordance with Danish data handling laws.
